# Effects of telehealth-integrated interventions for smoking cessation and alcohol reduction among people living with HIV: a systematic review and meta-analysis

**DOI:** 10.3389/fpubh.2026.1962757

**Published:** 2026-10-23

**Authors:** Ping Yang, Junbo Yi, Guli Asai, Fei Zhao

**Affiliations:** 1 School of Medicine, Northwest Minzu University, Lanzhou, Gansu, China; 2 Third Hospital of Shanxi Medical University, Shanxi Bethune Hospital, Shanxi Academy of Medical Sciences, Tongji Shanxi Hospital, Taiyuan, China; 3 Key Laboratory of Stomatology of State Ethnic Affairs Commission, Northwest Minzu University, Lanzhou, Gansu, China

**Keywords:** alcohol consumption, meta-analysis, people living with HIV, smoking cessation, systematic review, telehealth

## Abstract

**Background:**

Smoking and alcohol use are highly prevalent among people living with HIV (PLWH) and contribute to adverse health outcomes. Telehealth offers an accessible and scalable approach to behavioral support. However, previous reviews have generally focused on smoking or alcohol use separately. This systematic review and meta-analysis evaluated the effects of telehealth-integrated interventions on smoking cessation and alcohol-related outcomes among PLWH.

**Methods:**

PubMed, Embase, Cochrane Library, and Web of Science were searched from inception to April 1, 2026, for randomized controlled trials (RCTs) of interventions targeting tobacco or alcohol use among PLWH. Eligible studies were quantitatively synthesized, and the certainty of evidence for four main outcomes was assessed using GRADE.

**Results:**

Fifteen RCTs involving 2,212 PLWH were included. Compared with study-specific comparator conditions, telehealth-integrated intervention packages were associated with higher 7-day point-prevalence abstinence (RR = 2.07, 95% CI: 1.60–2.68) and 30-day point-prevalence abstinence (RR = 2.04, 95% CI: 1.31–3.19). For alcohol-related outcomes, limited evidence suggested that telehealth-integrated interventions may reduce mean drinks per drinking day (MD = −0.33, 95% CI: −0.58 – −0.08), whereas no clear between-group difference was observed in the number of drinking days in the past 30 days (MD = −0.02, 95% CI: −0.35 – 0.30). Exploratory subgroup analysis showed a larger smoking-cessation effect estimate at shorter follow-up (≤3 months) than at longer follow-up (>3 months) (*p* for subgroup difference = 0.02). No statistically significant difference in effect estimates was detected across telehealth modalities (*p* = 0.45), and this finding should not be interpreted as evidence of equivalent efficacy.

**Conclusion:**

Current evidence suggests that telehealth-integrated interventions may improve short-term smoking cessation outcomes among PLWH and may reduce mean drinks per drinking day. However, evidence for alcohol-related outcomes remains limited. Further adequately powered RCTs with longer follow-up are needed to clarify the durability of smoking cessation benefits and alcohol-related effects.

**Systematic Review Registration:**

https://www.crd.york.ac.uk/PROSPERO/view/CRD420251242171. Registration number: CRD420251242171)

## 1 Introduction

Tobacco use and alcohol consumption are two important, modifiable risk factors for adverse health outcomes among people living with human immunodeficiency virus (PLWH) (1, 2). Existing studies suggest that the current smoking rate among PLWH is around 36.1%, which is 2 to 3 times higher than that of the general population (3, 4). With the widespread adoption of antiretroviral therapy (ART), the risk of immunodeficiency-related deaths caused by HIV infection has decreased substantially (5). However, the all-cause mortality risk among PLWH remains notably higher than that of age- and sex-matched healthy individuals (6). Higher levels of tobacco and alcohol exposure are major contributors to the increased mortality risk in this population (1). Specifically, long-term exposure to tobacco and alcohol can offset the survival benefits of ART (1) and heighten the risk of cardiovascular disease, liver damage, and various types of cancer (7). Furthermore, smoking and drinking often co-occur among PLWH (7), and their synergistic effects exacerbate immunosuppression, accelerate HIV infection, and significantly amplify the risk of all-cause mortality (2).

Although it is widely recognized that smoking and alcohol consumption severely harm the physical and mental health of PLWH, there remain multiple barriers to interventions targeting tobacco and alcohol use among this population (3). Regarding smoking, medication combined with behavioral interventions can only achieve short-term smoking cessation and cannot sustain long-term benefits (8). Regarding harmful drinking, although various clinical guidelines recommend comprehensive intervention programs combining medication with psychological and behavioral approaches, this paradigm remains extremely underutilized in real-world clinical settings, and the forms and implementation pathways of these interventions urgently need optimization. Even though traditional behavioral interventions can improve harmful smoking and drinking behaviors among PLWH (8), interventions relying on in-person clinic visits still face multiple objective limitations. Primarily, offline medical visits carry a risk of identity exposure, and the resulting stigma weakens patients’ willingness to engage. Additionally, frequent trips to the clinic entail considerable time costs. Compounded by the uneven distribution of intervention resources across regions, these multiple constraints substantially reduce patients’ sustained adherence to the interventions. Therefore, exploring new alternative interventions better suited to the actual needs of PLWH carries significant practical implications.

Existing research confirms that telehealth can overcome various objective limitations inherent in traditional in-person interventions and holds promise as an alternative for behavior modification regarding tobacco and alcohol use among PLWH (8). Telehealth refers to remote health management and behavioral intervention services delivered via information and communication technology, including telephone consultations, text messages, online web platforms, and smartphone applications (apps) (9). It offers a new intervention approach for modifying tobacco and alcohol-related harmful behaviors among PLWH (8). Compared with traditional in-person interventions, telehealth offers greater accessibility, better privacy protection, high cost-effectiveness, and greater scalability (10). Relevant clinical trials have also corroborated the practical value of telehealth interventions. A pragmatic randomized controlled trial (RCT) in Vietnam, covering 13 HIV outpatient clinics, confirmed that interventions for tobacco cessation delivered via telehealth were clinically feasible (11). Telehealth interventions for harmful drinking have also yielded significant results. Incorporating supportive text messages into these interventions can effectively enhance abstinence maintenance rates and reduce patients’ total alcohol consumption (12).

Based on the available clinical evidence, multimodal interventions incorporating telehealth components show potential for clinical application in managing tobacco and alcohol use among PLWH. However, most published systematic reviews to date have focused solely on either smoking or drinking as a single addictive behavior. Very few studies have simultaneously integrated intervention evidence on interventions for both tobacco and alcohol use within a single systematic review framework and conducted quantitative meta-analyses. To address these limitations, this study simultaneously included smoking cessation and alcohol-related outcomes and systematically and quantitatively evaluated the effects of interventions incorporating telehealth components on smoking cessation and alcohol reduction among PLWH, with the aim of providing evidence-based guidance for the concurrent management of tobacco and alcohol use in this population.

## 2 Methods

This paper was prospectively registered with PROSPERO (identifier: CRD420251242171; URL: https://www.crd.york.ac.uk/PROSPERO/view/CRD420251242171) (13), and all reporting adhered to the PRISMA 2020 guidelines (14).

### 2.1 Search strategy and literature screening

PubMed, EMBASE, the Cochrane Library, and Web of Science were searched from database inception to April 1, 2026. Key search terms included “HIV,” “AIDS,” “telehealth,” “mHealth,” “eHealth,” “digital,” “smartphone,” “SMS,” “smoking cessation,” “alcohol abstinence,” and “randomized controlled trial,” among others. The search combined controlled subject headings with free-text terms. The specific search strings used for each database are presented in Appendix 1. Two researchers (Ping Yang and Junbo Yi) independently screened the literature according to a predefined search strategy. Following automatic deduplication via Zotero, two investigators independently screened titles and abstracts as per the predefined eligibility criteria. The potentially relevant records were subsequently evaluated in full text. For any disagreements, a third reviewer (Fei Zhao) was consulted to jointly determine whether to include it.

### 2.2 Eligibility criteria

Inclusion criteria covered (1) publicly published RCTs; (2) study participants were HIV-infected individuals aged ≥18 years (diagnosed via HIV antibody/antigen or nucleic acid testing), and current smokers (smoking ≥ 5 cigarettes per day or having smoked within the past 30 days) or current heavy drinkers (meeting the DSM-IV or DSM-5 diagnostic criteria for alcohol use disorder, or engaging in binge drinking defined as men consuming ≥ 5 drinks at a time, for women, ≥ 4 drinks on a single occasion) at baseline; (3) the intervention group received an intervention package incorporating telehealth components and directly targeting smoking or alcohol use, such as smartphone applications, text messaging, web-based platforms, instant messaging, or telephone-based support; these telehealth components could be combined with standard care, pharmacotherapy, or other behavioral support; (4) the control group received usual care, no intervention, pharmacotherapy, a non-telehealth behavioral intervention, or an attention-control condition; digital comparator conditions were eligible when designed primarily for general health education, attention matching, or nonspecific health promotion rather than as an active telehealth intervention targeting the same behavior; (5) smoking cessation rates or alcohol-related behavioral outcomes assessed using biochemical tests, standardized assessment instruments (such as the Timeline Followback [TLFB]), or structured questionnaires were clearly reported.

Exclusion criteria included: (1) non-RCT designs (observational studies, case reports), non-primary literature (reviews, commentaries, letters), unpublished or preliminary outputs (conference abstracts, protocols), and animal research; (2) participants had severe mental and cognitive impairments or other serious physical complications; (3) using electronic devices solely for collecting behavioral data without active behavioral interventions (e.g., monitoring smoking or drinking behavior without providing counseling, feedback, or other interventions); (4) the full-text literature was unavailable or valid outcome data could not be extracted; (5) duplicate studies.

### 2.3 Assessment of risk of bias

The risk of bias was appraised independently by two researchers via the Cochrane Risk of Bias Assessment Tool (ROB 2.0) (15). This instrument encompasses five distinct domains: the randomization process, deviations from the assigned intervention, incomplete outcome data, the method of outcome assessment, and selective outcome reporting. For every domain, the two assessors independently assigned one of the three ratings: low risk, some concerns, or high risk. Any inconsistencies were addressed through dialog, with unresolved conflicts adjudicated by a third researcher.

### 2.4 Data extraction

Two researchers (Ping Yang and Junbo Yi) independently extracted relevant information from the included RCTs and cross-checked their results. Any discrepancies were settled through discussion and negotiation; if they could not reach a consensus, a third researcher was consulted for adjudication. The primary information extracted included the first author, publication year, country, study design, baseline characteristics of the study population (sample size, age, and sex distribution), follow-up time point, specific components of the intervention and comparator groups, and outcome measures. To characterize the actual treatment contrast between study arms, we further extracted the complete intervention and comparator components, including telehealth delivery modality, pharmacotherapy, counselor-delivered or other behavioral support, face-to-face components, and other co-interventions. For smoking cessation studies, 7-day and 30-day point-prevalence abstinence rates were extracted; for alcohol-related studies, mean drinks per drinking day and the number of drinking days in the past 30 days were extracted. Methods used to assess or verify the outcomes were also recorded, including biochemical verification for smoking outcomes and standardized assessment instruments for alcohol-related outcomes. When the same outcome was reported at multiple follow-up assessments, only one effect estimate per study was included in a given meta-analysis, and the longest follow-up at which the relevant outcome was available was selected for the primary quantitative synthesis. Follow-up time points were standardized and reported in months. The 7-day and 30-day point-prevalence abstinence outcomes referred to abstinence during the 7 or 30 days immediately preceding the selected follow-up visit, rather than outcomes assessed on day 7 or day 30 after randomization. For smoking cessation outcomes, intention-to-treat (ITT) data were preferentially extracted when available, following the original study’s approach to handling missing outcome data. When an ITT estimate could not be extracted or reconstructed and only complete-case or available-case results were reported, the corresponding extractable results from the original study were used.

### 2.5 Statistical analysis

All quantitative syntheses were performed with Stata 19.5. For the 7-day and 30-day point prevalence abstinence rates, effect sizes were depicted as relative risk (RR) along with the corresponding 95% confidence interval (CI). To facilitate clinical interpretation, pooled risk differences (RDs) with corresponding 95% CIs were additionally calculated for the smoking cessation outcomes using the same fixed-effect framework. Where the pooled RD indicated benefit, the number needed to treat for benefit (NNTB) was calculated as the reciprocal of the absolute risk difference. Mean differences (MD) with corresponding 95% CI were used for mean drinks per drinking day and the number of drinking days in the past 30 days. The I^2^ was used to assess statistical heterogeneity. For multi-arm trials with a shared comparator, relevant intervention arms were combined for the overall meta-analysis. When intervention arms contributed to different modality subgroups, the sample size and event count of the shared comparator were split across comparisons to avoid double-counting participants. Given the generally low statistical heterogeneity observed in the primary analyses, fixed-effect models were used for the primary quantitative syntheses. In view of the clinical heterogeneity across studies in intervention components, comparator conditions, and follow-up time point, random-effects sensitivity analyses were additionally conducted for the main outcomes to assess the robustness of the pooled estimates to model assumptions.

For outcomes including at least 10 studies, potential publication bias was assessed using funnel plots and Begg’s test (16). Formal assessment of publication bias was not performed for outcomes including fewer than 10 studies. Sensitivity analyses included leave-one-out analyses, random-effects re-analysis of the main outcomes, and, where applicable, exclusion of studies rated at high risk of bias for the respective outcome.

Exploratory subgroup analyses were conducted according to intervention modality and follow-up time point to examine possible differences in effect estimates across study-level characteristics. Statistical significance was defined as *p* < 0.05 for between-group differences. Exploratory univariable meta-regression analyses were conducted for the 7-day point-prevalence abstinence outcome. Models were fitted using restricted maximum likelihood estimation, with standard errors corrected using the Knapp-Hartung method. Mean age, proportion of male participants, and total sample size were separately entered as study-level covariates in the univariable meta-regression analyses to explore possible associations between these study characteristics and the estimated intervention effects. Given the limited number of studies, these analyses were considered exploratory and hypothesis-generating rather than confirmatory assessments of effect modification.

### 2.6 Certainty of evidence assessment

The certainty of evidence for each main outcome was assessed using the Grading of Recommendations Assessment, Development and Evaluation (GRADE) approach. Evidence from RCTs initially started at high certainty and was evaluated across five domains: risk of bias, inconsistency, indirectness, imprecision, and publication bias. Risk of bias was informed by the RoB 2 assessment; inconsistency was evaluated considering statistical heterogeneity and the consistency of effect estimates; indirectness was assessed according to the relevance of the population, interventions, comparators, and outcomes; and imprecision was assessed based on the width of the 95% CI and the available information size. For outcomes including at least 10 studies, potential publication bias was assessed using funnel plots and Begg’s test; for outcomes including fewer than 10 studies, formal statistical assessment of publication bias was not performed, although potential publication bias was considered qualitatively in the GRADE assessment. The overall certainty of evidence was categorized as high, moderate, low, or very low.

## 3 Results

### 3.1 Literature screening

The initial literature search identified 2,161 records: 982 from PubMed, 228 from Embase, 405 from the Cochrane Library, and 546 from Web of Science. Following removal of 311 duplicate records, 1,850 records remained. Following an initial screening of titles and abstracts, 1,813 records were excluded for irrelevant topics (*n* = 212), interventions not involving telehealth (*n* = 445), study participants not meeting inclusion criteria (*n* = 739), ineligible document types such as reviews and conference abstracts (*n* = 212), irrelevant outcome measures (*n* = 196), and lack of a control group (*n* = 9). The remaining 37 articles proceeded to full-text re-screening. Among these, three articles were excluded because only abstracts were available and full texts could not be obtained. The full texts of the remaining 34 articles were reviewed, and 19 were removed for non-RCTs (*n* = 6), study participants not infected with HIV (*n* = 3), lacking required outcome data (*n* = 8), and interventions that did not meet the inclusion criteria (*n* = 2). Ultimately, 15 articles were included for quantitative synthesis. The literature screening process is presented in Figure 1.

**Figure 1:**
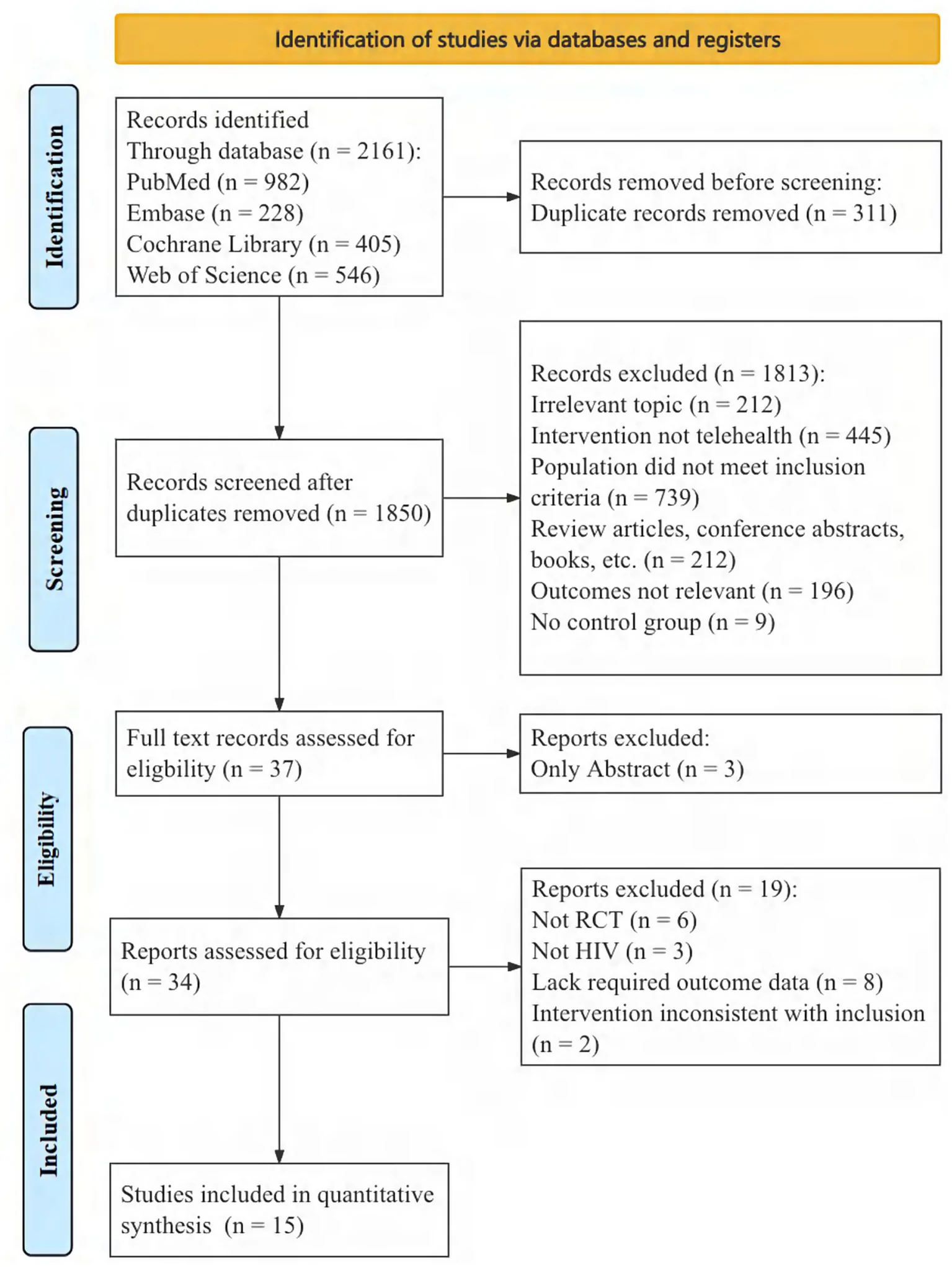
Flow diagram of literature screening. Adapted from the PRISMA 2020 flow diagram (14), licensed under CC BY 4.0.

### 3.2 Study features

Of the 15 RCTs, 13 were from the United States, 1 from China, and 1 from Cambodia. The total number of study participants was 2,212, with 1,119 in the intervention group and 1,093 in the control group. Specifically, in the smoking subgroup, there were 941 participants in the intervention group and 919 in the control group; in the alcohol consumption subgroup, there were 178 participants in the intervention group and 174 in the control group. Regarding methodological characteristics, two RCTs explicitly reported a multicenter design (17, 18), and eight RCTs (12, 17–23) reported clinical trial registration information. Regarding the type of study participants, 11 RCTs focused on HIV-infected smokers, 10 of which involved participants who smoked ≥ 5 cigarettes per day; four RCTs focused on HIV-infected heavy drinkers or individuals with alcohol use disorder.

Eleven RCTs on telehealth interventions for PLWH who smoked were included and categorized into five types based on intervention modalities: (1) telephone interventions (*n* = 3), which involved 8–11 proactive telephone follow-ups and implemented smoking cessation interventions based on cognitive behavioral therapy and motivational interviewing. Some studies also included a counseling hotline; (2) Web-based interventions (*n* = 3), which established online platforms for smoking cessation courses for participants’ self-directed learning; some incorporated online communities; (3) text message interventions (*n* = 3), centered on automated message delivery and covering smoking cessation techniques, behavioral reminders, and adherence prompts; some offered supplementary basic online support; (4) mobile app interventions (*n* = 1), including comprehensive smoking cessation software and mobile web platforms; the video-observed therapy module had low acceptance due to privacy concerns. Most smoking cessation interventions were used in conjunction with nicotine replacement therapy or varenicline; (5) telephone combined with text message (*n* = 2), centered on regular, thorough telephone counseling, supplemented by automated text messages for smoking cessation reminders and medication adherence prompts, combining human intervention with high-frequency outreach to enhance effectiveness.

Four additional interventions targeted alcohol use among PLWH. Three of these (18, 22, 24) utilized the HealthCall platform, available in both an interactive voice response and a mobile app. They implemented interventions through daily behavior monitoring and monthly visual feedback. The core techniques were motivational interviewing and clinical guidance. The remaining study (12) consisted of a single in-person session of cognitive behavioral therapy combined with a 12-week bidirectional text messaging intervention, which included medication reminders and counselor call-out features. None of the included alcohol-related interventions provided alcohol-related adjunctive medications. Details are outlined in Table 1.

**Table 1:** Basic characteristics of included studies.

Study	Country	Sample size (n)	Age (years, mean ± SD)	Sex (n)	Population	Intervention	Control	Follow-up time point	Outcome Measures	Verification Method
		I/C	I/C	M/F						
Vidrine (2006) (25)	USA	48/47	42.6 ± 8.2/43.1 ± 8.1	74/21	HIV + smokers (>5 cigarettes/day, CO ≥ 7 ppm)	Cell phone counseling (8 sessions) + NRT	Standard care (brief advice + self-help materials + NRT)	3 months	7-day PPA	ECO < 7 ppm
Vidrine (2012) (26)	USA	236/238	43.9 ± 8.3/45.7 ± 7.8	332/142	HIV + smokers (≥5 cigarettes/day, CO ≥ 7 ppm)	Cell phone counseling (11 sessions) + NRT	Standard care (brief advice + self-help materials + NRT)	3 months	7-day PPA + 30-day PPA	ECO < 7 ppm
Humfleet (2013) (27)	USA	58/82	–	–	HIV + smokers (smoke most days)	Computer-based Internet intervention (CBI) + NRT	Self-help (NRT only)	12 months	7-day PPA	ECO ≤ 10 ppm
Shuter (2014) (28)	USA	69/69	45.4 ± 9.9/45.6 ± 9.9	76/60	HIV + smokers (interested in quitting within 6 months)	PSFW (web-based program) + NRT	Standard care (brief advice + self-help booklet + NRT)	3 months	7-day PPA	ECO < 10 ppm
Tseng (2017) (16)	USA	54 + 51/53	–	125/29	HIV + smokers (≥5 cigarettes/day, willing to quit within 2 weeks)	Text messaging + varenicline; Text messaging + telephone counseling + varenicline	Standard care (varenicline only)	3 months	7-day PPA	ECO < 8 ppm
Shuter (2020) (29)	USA	48/52	45.8 ± 10.0/–	53/47	HIV + smokers (interested in quitting within 6 months)	PSF-M (smartphone-based web + text messaging) + NRT	Standard care (brief advice + self-help booklet + NRT)	4 months	7-day PPA + 30-day PPA	ECO < 10 ppm
Bui (2022) (30)	Cambodia	25/25	44.7 ± 9.7/46.9 ± 10.8	50/0	HIV + smokers (≥5 cigarettes/day, willing to set a quit date)	Automated Messaging (AM) + standard care	Standard care (brief advice + self-help booklet)	2 months	7-day PPA	ECO < 8 ppm
Shuter (2022) (20)	USA	255/251	–	287/219	HIV + smokers (motivated to quit, planning to quit within 6 months)	PSFW+ (web-based program + online community) + NRT	attention control + NRT	6 months	7-day PPA + 30-day PPA	Expired CO < 10 ppm + salivary cotinine
Asfar (2025) (23)	USA	13/16	52.5 ± 10.0/52.6 ± 8.7	21/18	HIV + smokers (≥5 cigarettes/day, interested in quitting within 30 days)	Mindfulness Training (MT) app (Craving-to-Quit) ± Emocha + NRT	Standard care (brief advice + NRT + self-help booklet)	3 months	7-day PPA	ECO < 6 ppm
Cioe (2025) (21)	USA	30/34	56.2 ± 9.4/52.9 ± 12.0	38/26	HIV + smokers (≥5 cigarettes/day, positive cotinine)	Peer navigation telephone counseling (12 sessions) + nurse counseling	Standard care (nurse counseling)	6 months	7-day PPA	Expired CO < 10 ppm + salivary cotinine
Huang (2025) (19)	China	54/55	44.2 ± 14.5/46.3 ± 15.8	105/4	HIV + smokers (≥5 cigarettes/day, willing to set a quit date within 1 month)	WeChat-based messaging + NRT + behavioral counseling	NRT + self-help booklet	3 months	7-day PPA	ECO < 8 ppm
Hasin (2013) (18)	USA	75/78	45.7 ± 8.1/46.5 ± 7.9	124/39	HIV + heavy drinkers (≥4 drinks/occasion in the past 30 days)	Motivational Interviewing (MI) + HealthCall (IVR)	MI-only	2 months	drinks per drinking day	TLFB
Aharonovich (2017) (24)	USA	21/21	51.0 ± 7.0/50.9 ± 7.2	37/5	HIV + with non-injection drug use + binge drinking (≥4 drinks/occasion)	MI + HealthCall-S (smartphone app)	MI-only	2 months	drinks per drinking day + number of drinking days in the past 30 days	TLFB
Glasner (2020) (12)	USA	18/17	47.6 ± 11.5/52.7 ± 6.1	30/5	HIV + with alcohol use disorder (DSM-V)	CBT-based text messaging (12 weeks) + face-to-face CBT session	Informational pamphlet (INFO)	3 months	number of drinking days in the past 30 days	ASI + TLFB
Hasin (2022) (22)	USA	64/58	46.7 ± 10.7/46.5 ± 10.1	-	HIV + with alcohol dependence (DSM-IV, >4 drinks/occasion in the past 30 days)	Clinician’s Guide (CG) + HealthCall	CG-only	12 months	drinks per drinking day + number of drinking days in the past 30 days	TLFB

PPA, point prevalence abstinence; 7-day/30-day PPA, 7-day/30-day point prevalence abstinence; CO, carbon monoxide; ECO/exhaled CO; ppm, parts per million; TLFB, Timeline Followback; ASI, Addiction Severity Index; NRT, nicotine replacement therapy; MI, motivational interviewing; IVR, interactive voice response; CBI, computer-based intervention; PSFW, a web-based smoking cessation program for people living with HIV.

### 3.3 Quality assessment

The risk-of-bias assessments across domains are summarized in Figure 2A, while the study-specific judgments for each domain and overall risk of bias are presented in Figure 2B. Overall, three RCTs (20.0%) were classified as low risk, 10 (66.7%) had some concerns, and two (13.3%) were rated as high risk.

**Figure 2:**
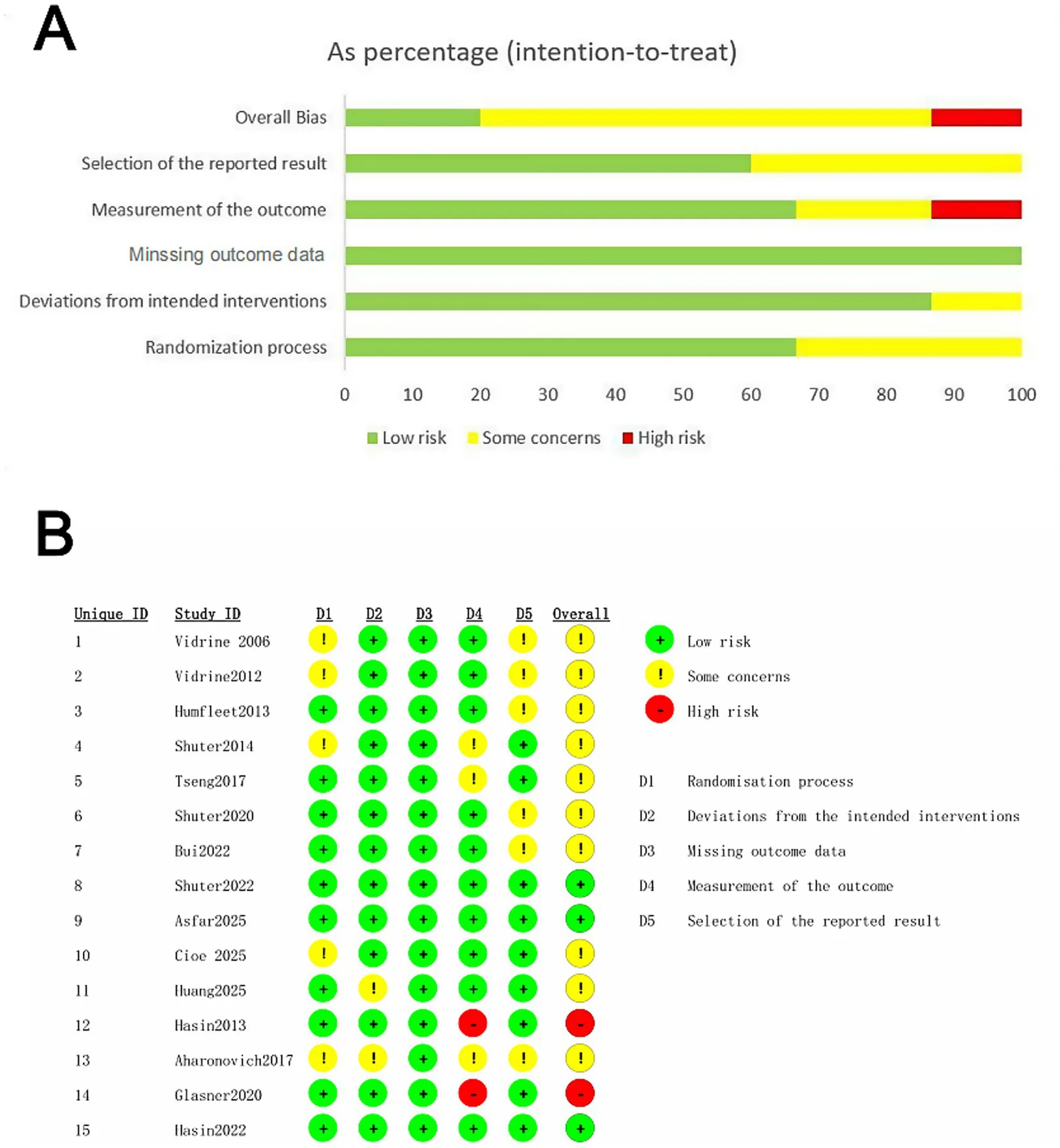
Risk of bias assessment of included trials using RoB 2.0. **(A)** Risk of bias graph; **(B)** risk of bias summary plot (traffic light plot).

Regarding the randomization process, 10 RCTs fully described the methods used to generate random sequences and conceal allocation and were classified as low risk. The remaining five RCTs merely noted the use of random allocation but provided no explicit description of the allocation concealment procedures. Consequently, these five trials were judged to raise some concerns regarding selection bias. Blinding of participants proved difficult owing to the inherent limitations of telehealth interventions, particularly regarding deviations from the intended protocol. However, most RCTs implemented the intervention according to protocol without substantial deviations from the protocol. Thirteen RCTs were classified as low risk, while two were classified as having some concerns due to intervention deviations that could potentially affect the outcomes. Regarding missing outcome data, the outcome data in all 15 RCTs were relatively complete, or the loss-to-follow-up rates were low and balanced across groups, introducing no apparent bias. All were classified as low risk. Regarding outcome measurement, 10 RCTs used objective biochemical indicators such as exhaled carbon monoxide and cotinine, or conducted standardized outcome assessments by blinded evaluators, and thus had a low risk of bias. Three RCTs relied solely on participant self-reports without objective biochemical tests, indicating a certain risk of measurement bias. Two RCTs relied entirely on participants’ subjective behavioral self-assessments, did not blind evaluators, and lacked validation by objective indicators, resulting in a high risk of subjective measurement bias. Regarding selective reporting of results, eight RCTs reported outcomes strictly in accordance with pre-registered protocols and analysis plans and were therefore assessed as low risk. The remaining seven RCTs, however, lacked publicly available clinical trial registration information or did not clearly report their analysis plans. Consequently, the possibility of selective reporting could not be eliminated, and these studies were assessed as presenting some concern.

The distribution of the proportion of studies across each bias risk dimension is shown in Figure 2A. Among these, the dimension of missing outcome data performed best, with all included RCTs classified as low risk. For the dimension of deviation from the intended intervention, 93.3% of studies were rated as low risk, whereas 66.7% achieved this rating regarding the randomization process. The dimensions of outcome measurement and selective reporting were the principal contributors to methodological bias. More precisely, a high risk of bias was noted in 13.3% of studies regarding outcome measurement, whereas merely 53.3% of trials met the low-risk threshold for selective reporting.

### 3.4 Meta-analysis results

#### 3.4.1 Effect of telehealth-integrated interventions on the 7-day point prevalence abstinence rate among PLWH

In the meta-analysis, 11 RCTs (16, 19–21, 23, 25–30) reported the 7-day point prevalence abstinence rate among PLWH.

A fixed-effect model using the Mantel–Haenszel method was used for the primary analysis, with low statistical heterogeneity observed across studies (*I*^2^ = 0.0%, *p* = 0.650). A random-effects model was additionally applied as a sensitivity analysis to account for clinical heterogeneity across studies. The meta-analysis results demonstrated that compared with the study-specific comparator conditions, telehealth-integrated intervention packages were associated with higher 7-day point-prevalence abstinence among PLWH (RR = 2.07, 95% CI: 1.60–2.68) (Supplementary Figure S1A). The corresponding pooled RD was 0.086 (95% CI: 0.057–0.115), representing an absolute increase of approximately 8.6 percentage points in 7-day point-prevalence abstinence and corresponding to an NNTB of approximately 12. Although overall statistical heterogeneity was low, exploratory subgroup analyses were conducted according to intervention modality and follow-up time point to examine possible differences in effect estimates across study-level characteristics. Given that the subgroup analyses were predefined exploratory analyses and the number of studies in each group was limited, the results should be carefully interpreted.

Exploratory subgroup analysis by intervention modality (Figure 3) showed higher 7-day point-prevalence abstinence rates for telephone interventions (RR = 2.99, 95% CI: 1.67–5.38), combined telephone and text-message interventions (RR = 2.57, 95% CI: 1.35–4.91), and web-based interventions (RR = 1.62, 95% CI: 1.12–2.35). In contrast, the pooled estimates for text-messaging interventions (RR = 1.95, 95% CI: 0.89–4.28) and app-based interventions (RR = 2.46, 95% CI: 0.76–7.99) were not statistically significant. No statistically significant difference in effect estimates was detected across intervention-modality subgroups (*p* for interaction = 0.45). Given the limited number of studies within individual subgroups, these findings should be interpreted cautiously and should not be considered evidence of equivalent efficacy across modalities.

**Figure 3:**
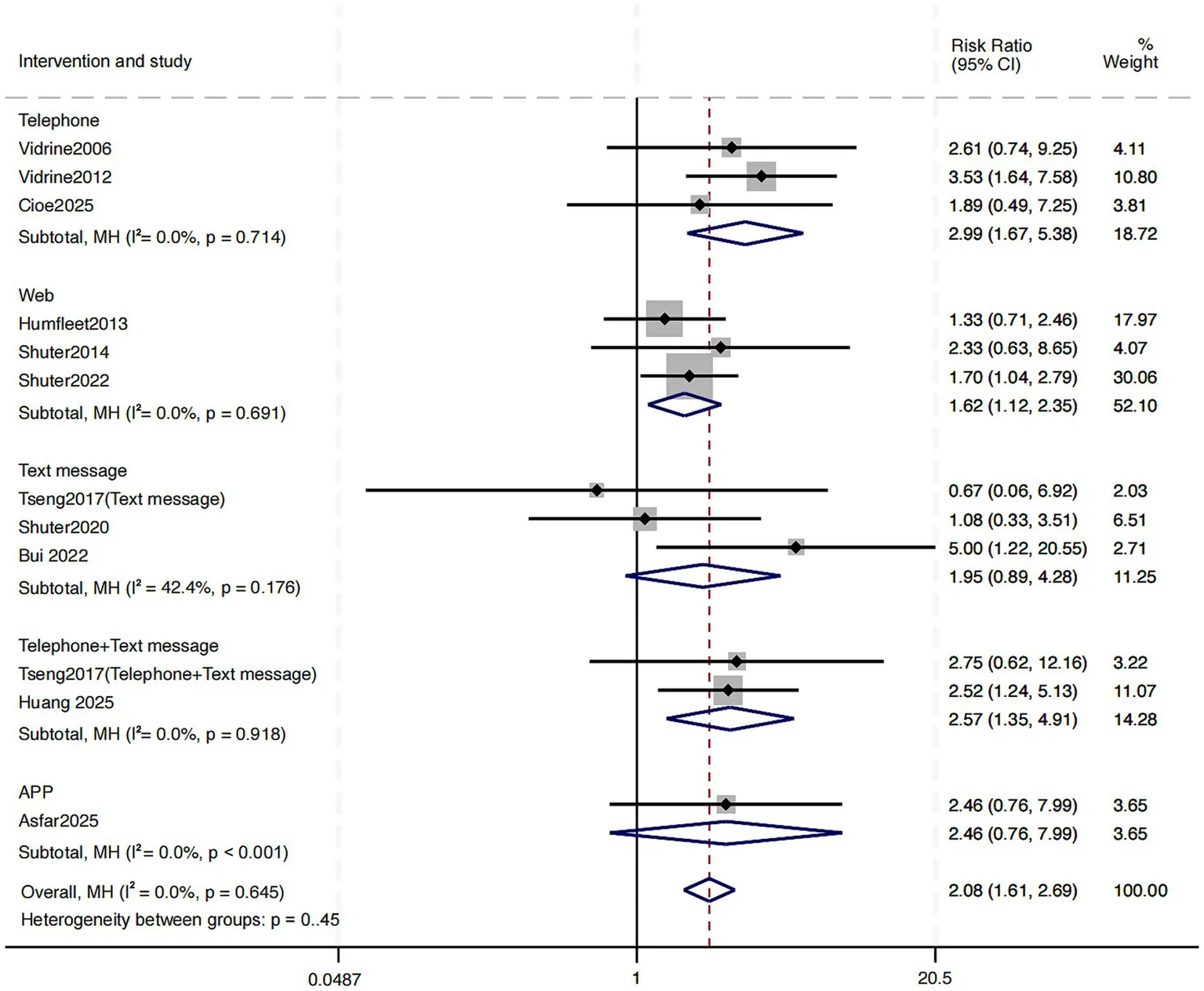
Subgroup analysis of the 7-day point prevalence abstinence rate stratified by intervention type.

In the exploratory subgroup analysis by follow-up time point (Figure 4), the effect estimates in both the short-term (≤3 months) and longer-term (>3 months) follow-up subgroups favored the intervention. The effect estimate was larger in the short-term follow-up subgroup (RR = 2.82, 95% CI: 1.92–4.14) than in the longer-term follow-up subgroup (RR = 1.53, 95% CI: 1.07–2.18), with a statistically significant difference between subgroups (*p* for interaction = 0.02). This exploratory finding suggests that the intervention effect estimate may be larger at shorter follow-up time points than at longer follow-up time points; however, because this was a study-level subgroup comparison, it should not be interpreted as evidence that intervention effectiveness necessarily declines over time.

**Figure 4:**
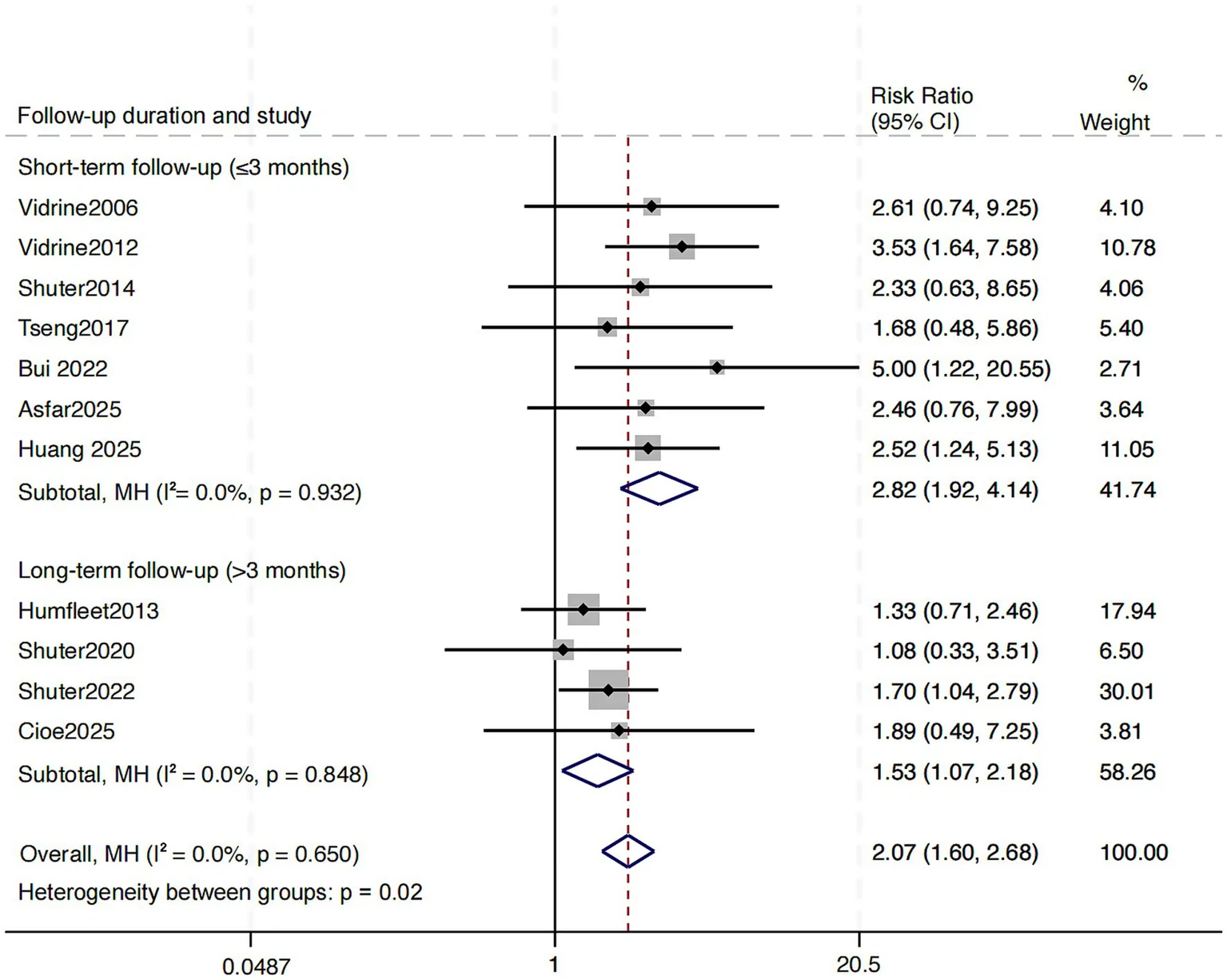
Subgroup analysis of the 7-day point prevalence abstinence rate stratified by follow-up time point.

#### 3.4.2 Effect of telehealth-integrated interventions on the 30-day point prevalence abstinence rate among PLWH

Three RCTs (20, 26, 29) reported the 30-day point prevalence abstinence rate (Figure 5A). A fixed-effect model was used for the primary analysis, with low statistical heterogeneity observed across studies (*I*^2^ = 0.0%, *p* = 0.480). Compared with the study-specific comparator conditions, telehealth-integrated interventions were associated with higher 30-day point-prevalence abstinence among PLWH (RR = 2.04, 95% CI: 1.31–3.19). The corresponding pooled RD was 0.052 (95% CI: 0.021–0.083), representing an absolute increase of approximately 5.2 percentage points in 30-day point-prevalence abstinence and corresponding to an NNTB of approximately 20.

**Figure 5:**
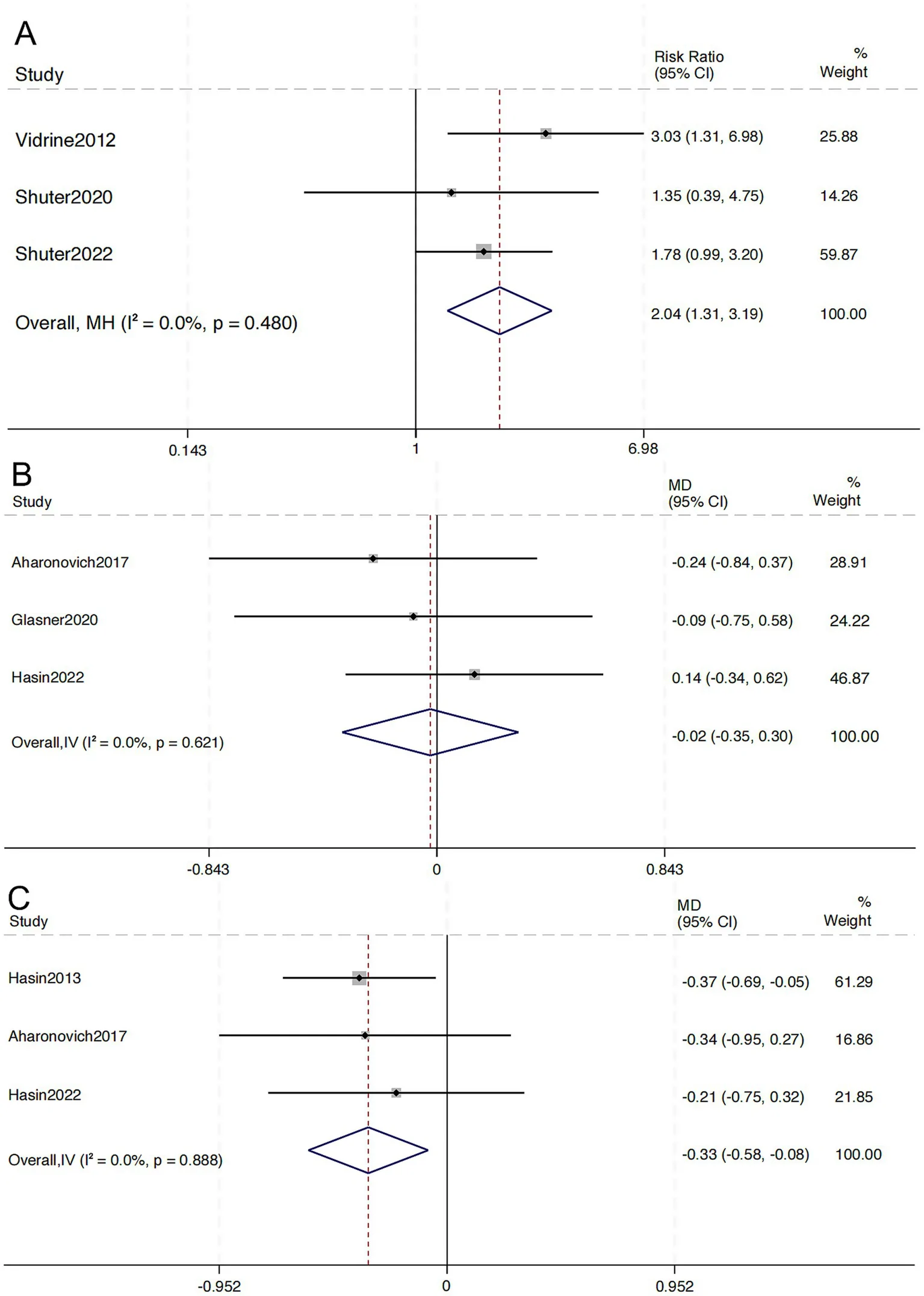
Pooled analysis using a fixed-effect model. **(A)** 30-day point prevalence abstinence rate; **(B)** number of drinking days in the past 30 days; **(C)** drinks per drinking day.

#### 3.4.3 Effect of telehealth-integrated interventions on the number of drinking days in the past 30 days among PLWH

Three RCTs (12, 22, 24) reported the number of drinking days in the past 30 days (Figure 5B). A fixed-effect model was used for the primary analysis, with low statistical heterogeneity observed across studies (*I*^2^ = 0.0%, *p* = 0.621). Compared with the study-specific comparator conditions, no clear between-group difference was observed in the number of drinking days in the past 30 days among PLWH (MD = −0.02, 95% CI: −0.35–0.30).

#### 3.4.4 Effect of telehealth-integrated interventions on mean drinks per drinking day among PLWH

Three studies (18, 22, 24) reported mean drinks per drinking day (Figure 5C). A fixed-effect model was used for the primary analysis, with low statistical heterogeneity observed across studies (*I*^2^ = 0.0%, *p* = 0.888). Compared with the study-specific comparator conditions, telehealth-integrated interventions were associated with a reduction in mean drinks per drinking day among PLWH (MD = −0.33, 95% CI: −0.58 – −0.08).

### 3.5 Publication bias and sensitivity analysis

For the 7-day point prevalence abstinence rate, which included 10 or more studies, this study plotted a funnel plot and conducted Begg’s test to assess publication bias (Figure 6A). The funnel plot appeared generally symmetrical, and Begg’s test yielded *p* = 0.186, providing no statistically significant evidence of publication bias. For the remaining three outcome measures, the number of studies included was less than 10, so no publication bias assessment was conducted. In the leave-one-out sensitivity analysis, the direction of the pooled effect remained generally stable, and sequential exclusion of individual studies did not materially alter the overall estimate (Figure 6B).

**Figure 6:**
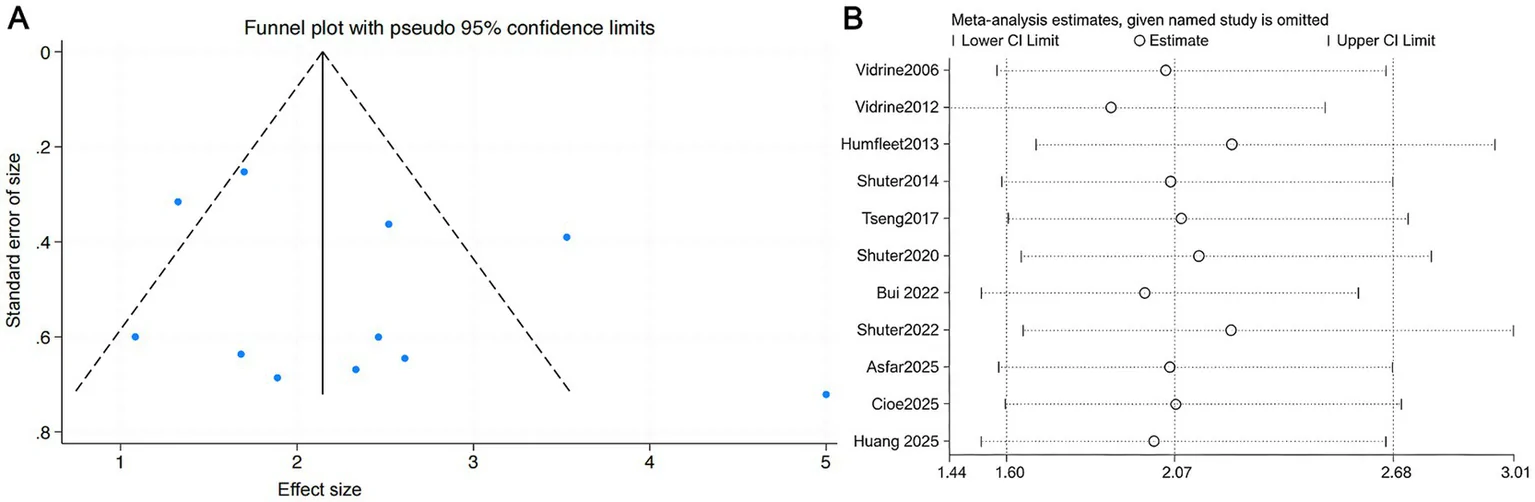
**(A)** A funnel plot for the 7-day point prevalence abstinence rate; **(B)** sensitivity analysis of the 7-day point prevalence abstinence rate.

Random-effects sensitivity analyses were also conducted for the four main outcomes to assess the robustness of the findings to model assumptions. For 7-day point-prevalence abstinence, the pooled estimate remained in favor of the intervention under the random-effects model (RR = 2.00, 95% CI: 1.54–2.59) (Supplementary Figure S1B). Similarly, the pooled estimate for 30-day point-prevalence abstinence remained materially unchanged (RR = 2.00, 95% CI: 1.28–3.14) (Supplementary Figure S2A). For the number of drinking days in the past 30 days, no clear between-group difference was observed under the random-effects model (MD = −0.02, 95% CI: −0.35 – 0.30) (Supplementary Figure S2B). For mean drinks per drinking day, the random-effects analysis continued to favor the intervention (MD = −0.33, 95% CI: −0.58 – −0.08) (Supplementary Figure S2C). Overall, the direction and interpretation of the findings were consistent with those of the primary fixed-effect analyses.

Sensitivity analyses excluding studies rated at high risk of bias for the respective alcohol-related outcome were also conducted. After exclusion of Glasner et al. (12), no clear between-group difference was observed in the number of drinking days in the past 30 days (MD = −0.00, 95% CI: −0.38 – 0.37; *I*^2^ = 0.0%), consistent with the primary analysis. For mean drinks per drinking day, exclusion of Hasin et al. (18) yielded an effect estimate in the same direction as the primary analysis, but the 95% CI crossed the null value (MD = −0.27, 95% CI: −0.67–0.13; *I*^2^ = 0.0%). The corresponding sensitivity analyses are presented in Supplementary Figure S3.

### 3.6 Meta-regression analysis

Exploratory univariable meta-regression analyses were conducted for the 7-day point-prevalence abstinence outcome to examine the associations between study-level mean age, proportion of male participants, total sample size, and the intervention effect expressed as log-transformed RR. Models were fitted using restricted maximum likelihood (REML) estimation with Knapp–Hartung adjustment of standard errors. Corresponding meta-regression scatter plots are presented in Supplementary Figure S4.

Mean participant age showed a negative association with log(RR) (*β* = −0.0300, 95% CI: −0.1200–0.0600; *p* = 0.4448) (Supplementary Figure S4A); whereas the proportion of male participants showed a positive association (*β* = 0.0124, 95% CI: −0.0018–0.0266; *p* = 0.0788) (Supplementary Figure S4B). No apparent association was observed between total sample size and log(RR) (*β* = 0.0001, 95% CI: −0.0014–0.0016; *p* = 0.9259) (Supplementary Figure S4C). None of the three study-level covariates was significantly associated with the intervention effect.

Given that only 11 RCTs were included and data for some covariates were available from fewer studies, the statistical power of the meta-regression analyses was limited. These findings should therefore be considered exploratory. The absence of statistically significant associations does not exclude potential moderating effects of these study-level characteristics, and further evidence from a larger number of primary studies is required.

### 3.7 Certainty of evidence

The certainty of evidence for the main outcomes was assessed using the GRADE approach. For 7-day point-prevalence abstinence (11 studies), no serious concerns were identified across the five GRADE domains, and the certainty of evidence was rated as high. For 30-day point-prevalence abstinence (3 studies), the certainty of evidence was rated as low because of imprecision related to the limited number of included studies and potential publication bias. For the number of drinking days in the past 30 days (3 studies), the 95% CI crossed the null value, indicating imprecision; in addition, formal assessment of publication bias could not be performed because of the limited number of studies. The certainty of evidence for this outcome was therefore rated as low. For mean drinks per drinking day (3 studies), the certainty of evidence was rated as low because of the limited number of included studies and potential publication bias. Detailed GRADE assessments are presented in Appendix 2.

## 4 Discussion

This study included 15 RCTs involving 2,212 PLWH and evaluated the effects of telehealth-integrated interventions on smoking and alcohol consumption among PLWH. The results indicated that telehealth-integrated interventions increased 7-day (RR = 2.07) and 30-day point-prevalence abstinence rates (RR = 2.04) among PLWH and reduced mean drinks per drinking day (MD = −0.33), while no clear between-group difference was observed in the number of drinking days in the past 30 days (MD = −0.02). Larger smoking cessation effect estimates were observed in studies with shorter follow-up (≤3 months). No statistically significant differences in effect estimates were detected across different intervention modalities. Exploratory meta-regression analyses detected no statistically significant study-level associations between the 7-day point-prevalence abstinence effect estimates and mean age, proportion of males, or study sample size.

Clinical heterogeneity existed in the composition of the included interventions. Telehealth components were frequently combined with pharmacotherapy, motivational interviewing, cognitive behavioral therapy, or other counselor-delivered behavioral support, while comparator conditions and other co-interventions also varied across trials. Accordingly, the present meta-analysis estimates the average incremental effect of intervention packages incorporating telehealth components relative to study-specific comparators, rather than the independent effect of telehealth technology, pharmacotherapy, or counseling. Because some comparator conditions included active pharmacological or behavioral components, the pooled estimates should not be interpreted as effects relative to no treatment; such active comparators may also reduce the observed between-group contrast.

Compared with systematic reviews targeting the general smoking population (31), this review observed a numerically larger effect estimate for smoking cessation at shorter follow-up among PLWH. At shorter follow-up (≤3 months), the 7-day point-prevalence abstinence rate was approximately 2.8 times that observed under the study-specific comparator conditions (RR = 2.82, 95% CI: 1.92–4.14). From a behavioral intervention perspective, telehealth-integrated smoking cessation interventions can use telephone calls, text messages, and mobile apps to provide low-barrier and frequent support. These tools can extend behavioral support beyond scheduled in-person visits and enable more frequent contact with participants (32).

Exploratory subgroup analysis indicated that the pooled effect estimate was larger in studies with shorter follow-up (≤3 months, RR = 2.82) than in those with longer follow-up (>3 months, RR = 1.53), with a statistically significant difference between subgroups (*p* = 0.02). This pattern may suggest that the magnitude of the observed intervention effect differs according to follow-up timing. However, because this was a study-level exploratory subgroup comparison, it does not establish that the effectiveness of telehealth-integrated interventions declines over time.

According to the Transtheoretical Model of behavior change (33), smoking cessation involves different stages of change, including the action and maintenance stages. In the early stages of smoking cessation, external reminders, such as text messages and phone calls, may help support behavioral change, which is consistent with the action phase, during which individuals actively modify their smoking behavior. However, long-term maintenance of smoking cessation may also require sustained self-efficacy for quitting and establishing positive, healthy behavioral patterns to replace smoking. These considerations may also be relevant to PLWH who smoke. Routine HIV care and ART follow-up may provide opportunities to integrate telehealth-based smoking cessation support into ongoing care. However, reduced self-regulatory capacity and continued exposure to stress or smoking-related cues may increase the risk of relapse, highlighting the potential importance of sustained support during smoking cessation maintenance (34).

Among the different intervention modalities, numerically larger effect estimates were observed in studies using telephone counseling (RR = 2.99) and combined telephone plus text messaging (RR = 2.57), whereas the effect estimate for app-based interventions also favored the intervention but its 95% CI crossed the null value (RR = 2.46, 95% CI: 0.76–7.99). However, no statistically significant difference in effect estimates was detected across intervention modalities. This finding should not be interpreted as evidence of equivalent efficacy across modalities. This result should not be interpreted as evidence that app-based interventions are ineffective, as the app subgroup included only one smoking cessation study and adherence to the app was low. As reported by Shuter et al. (29), only 9.5% of participants used the intervention app daily and consistently. In contrast, telephone counseling can provide direct human interaction and repeated contact, which may help sustain participants’ motivation. However, considering scalability, app and text message interventions still offer irreplaceable advantages in resource-limited settings, and their optimized application among PLWH warrants further exploration.

Findings for alcohol-related outcomes differed according to the specific outcome assessed. Limited evidence suggested that telehealth-integrated interventions may reduce mean drinks per drinking day (MD = −0.33), whereas no clear between-group difference was observed in the number of drinking days in the past 30 days (MD = −0.02). A broadly similar pattern was reported by Hasin et al. (18), with treatment effects observed for drinks per drinking day but not for the percentage of days abstinent during the previous 30 days. The evidence base for alcohol-related outcomes remained limited: only four alcohol-intervention RCTs involving 352 participants were identified, and each pooled alcohol outcome was based on only three studies, limiting statistical precision and power. In addition, alcohol-related outcomes were based on self-report and were not biochemically verified. Moreover, after exclusion of the study rated at high risk of bias for mean drinks per drinking day, the effect estimate remained in the same direction but the 95% CI crossed the null value, indicating some sensitivity of this finding to risk of bias. These findings should therefore be interpreted cautiously, and further adequately powered RCTs with objective corroboration of alcohol use, where feasible, are needed.

In terms of the quality of the included RCTs, the overall methodological quality was moderate, with biases primarily arising from subjective reporting of outcomes and non-standardized trial registration. Therefore, sensitivity analyses were conducted to assess robust conclusions. The limitations may provide insights for future research. Additional clinical trials are needed in regions with a high disease burden; trial registration processes should be standardized; drinking behavior should be assessed in conjunction with objective biochemical markers; and large-sample original studies should be included to strengthen subgroup evidence.

This study still has several limitations. First, geographical representativeness is insufficient. Of the 15 original RCTs included, 13 were conducted in the United States, and only one each in China and Cambodia. The lack of original studies from regions with a high HIV burden, such as Africa, other parts of Asia, and South America, limits the generalizability of this study’s conclusions to different geographic regions and health system contexts. Second, regarding outcome measurement, seven RCTs did not provide clinical trial registration information, and their trial protocol disclosures were incomplete, making it difficult to fully assess the potential risk of selective outcome reporting. Third, alcohol-related outcomes were based on self-report and were not biochemically verified, which may have affected the accuracy of the effect estimates. Finally, the number of studies available for the alcohol-related outcomes was small, with only three studies contributing to each pooled analysis, limiting statistical precision and power.

Despite the aforementioned limitations, this study still offers certain improvements and innovations compared to previous systematic reviews. Most existing reviews have analyzed only a single behavior—either smoking or drinking—in isolation. Some reviews did not restrict their analysis to RCT designs and did not use biochemical markers to verify smoking cessation. In contrast, this study simultaneously synthesized evidence for interventions targeting both behaviors. All included studies were RCTs, and smoking cessation was primarily assessed through biochemical tests, thereby elevating evidence strength compared to previous reviews. Notably, while the current evidence suggests that telehealth-integrated smoking cessation interventions may be beneficial for PLWH, the evidence base for alcohol intervention is insufficient to support definitive conclusions. More high-quality original studies are needed, particularly evidence from regions outside the United States.

## 5 Conclusion

This meta-analysis indicates that telehealth-integrated interventions may improve short-term smoking cessation outcomes among PLWH and may reduce mean drinks per drinking day. Exploratory subgroup analyses did not detect statistically significant differences in effect estimates across telehealth modalities, although this should not be interpreted as evidence of equivalent efficacy; larger smoking cessation effect estimates were observed in studies with shorter follow-up (≤3 months). Due to geographical bias in the included RCTs and the lack of objective biochemical indicators for alcohol consumption outcomes, the generalizability of these conclusions is limited. Further high-quality, adequately powered RCTs with longer follow-up are needed, particularly in high-HIV-burden settings that are currently underrepresented, with objective corroboration of alcohol use where feasible.

## Data Availability

The original contributions presented in the study are included in the article/Supplementary material, further inquiries can be directed to the corresponding author.

## Supplementary material

SUPPLEMENTARY FIGURE S1 Forest plot of the 7-day point prevalence abstinence rate. **(A)** The fixed-effect model; **(B)** the random-effects model.

SUPPLEMENTARY FIGURE S2 Pooled analysis using a random-effects model. **(A)** 30-day point prevalence abstinence rate; **(B)** number of drinking days in the past 30 days; **(C)** drinks per drinking day.

SUPPLEMENTARY FIGURE S3 Sensitivity analysis after excluding studies with high risk of bias for alcohol-related outcomes.

SUPPLEMENTARY FIGURE S4 Scatter plots showing the associations between study-level characteristics and the log relative risk of smoking abstinence. **(A)** Mean participant age, **(B)** proportion of male participants, and **(C)** total sample size per study.
