# Supplementary material for: Effects of telehealth-integrated interventions for smoking cessation and alcohol reduction among people living with HIV: a systematic review and meta-analysis
Source: Front Public Health. 2026 Oct 23;14:1962757. doi: 10.3389/fpubh.2026.1962757 (PMC13739805; doi:10.3389/fpubh.2026.1962757)
Supplement: Supplementary file 1 [file Data_Sheet_1.DOCX]

**Appendix 1:** Detailed search strategies and results for PubMed, EMBASE, Cochrane Library, and Web of Science.

| Pubmed | | |
| --- | --- | --- |
| # | Query | Results |
| 1 | "Acquired Immunodeficiency Syndrome"[Mesh] | 79,696 |
| 2 | "HIV"[Mesh] | 111,372 |
| 3 | #1 OR #2 | 179,742 |
| 4 | immunodeficiency associated virus[Title/Abstract] OR Human Immunodeficiency Viruses[Title/Abstract] OR human immunodeficiency virus infection[Title/Abstract] OR Human immuno deficiency virus[Title/Abstract] OR HIV[Title/Abstract] OR aquired immunodeficiency syndrome[Title/Abstract] OR aquired immune deficiency syndrome[Title/Abstract] OR AIDS Viruses[Title/Abstract] OR aids related virus[Title/Abstract] OR aids associated virus[Title/Abstract] OR aids associated retrovirus[Title/Abstract] OR aids associated lentivirus[Title/Abstract] OR AIDS[Title/Abstract] OR acquired immunodeficiency virus syndrome[Title/Abstract] OR Acquired Immuno-Deficiency Syndromes[Title/Abstract] OR Acquired Immunodeficiency Syndromes[Title/Abstract] OR Acquired Immunodeficiency Syndrome Virus[Title/Abstract] OR acquired immunodeficiency disease syndrome[Title/Abstract] OR Acquired Immune Deficiency Syndrome Virus[Title/Abstract] OR Acquired Immune Deficiency Syndrome[Title/Abstract] OR acquired immune deficiency disease syndrome[Title/Abstract] OR acquired human immunodeficiency syndrome[Title/Abstract] | 485,615 |
| 5 | #3 OR #4 | 507,028 |
| 6 | Smok*[Title/Abstract] OR drink*[Title/Abstract] OR alcohol[Title/Abstract] | 785,869 |
| 7 | #5 AND #6 | 15,471 |
| 8 | randomized controlled trial[Title/Abstract] OR controlled clinical trial[Title/Abstract] OR placebo[Title/Abstract] OR random allocation[Title/Abstract] OR randomized[Title/Abstract] | 929,204 |
| 9 | #7 AND #8 | 982 |

| Embase | | |
| --- | --- | --- |
| # | Query | Results |
| 1 | 'acquired immune deficiency syndrome'/exp | 608587 |
| 2 | 'human immunodeficiency virus'/exp | 234456 |
| 3 | #1 OR #2 | 796168 |
| 4 | 'immunodeficiency associated virus':ab,ti OR 'human immunodeficiency viruses':ab,ti OR 'human immunodeficiency virus infection':ab,ti OR 'human immuno deficiency virus':ab,ti OR hiv:ab,ti OR 'aquired immunodeficiency syndrome':ab,ti OR 'aquired immune deficiency syndrome':ab,ti OR 'aids viruses':ab,ti OR 'aids related virus':ab,ti OR 'aids associated virus':ab,ti OR 'aids associated retrovirus':ab,ti OR 'aids associated lentivirus':ab,ti OR aids:ab,ti OR 'acquired immunodeficiency virus syndrome':ab,ti OR 'acquired immuno-deficiency syndromes':ab,ti OR 'acquired immunodeficiency syndromes':ab,ti OR 'acquired immunodeficiency syndrome virus':ab,ti OR 'acquired immunodeficiency disease syndrome':ab,ti OR 'acquired immune deficiency syndrome virus':ab,ti OR 'acquired immune deficiency syndrome':ab,ti OR 'acquired immune deficiency disease syndrome':ab,ti OR 'acquired human immunodeficiency syndrome':ab,ti | 632898 |
| 5 | #3 OR #4 | 1105502 |
| 6 | smok*:ab,ti OR drink*:ab,ti OR alcohol:ab,ti | 1140594 |
| 7 | #5 AND #6 | 34118 |
| 8 | 'randomized controlled trial':ab,ti OR 'controlled clinical trial':ab,ti OR placebo:ab,ti OR 'random allocation':ab,ti OR randomized:ab,ti | 1538730 |
| 9 | #7 AND #8 | 2100 |
| 10 | 'digital or telephone or telemedicine or internet or web or application ormessage':ab,ti OR phone:ab,ti OR mobile:ab,ti OR remote:ab,ti OR website:ab,ti | 454749 |
| 11 | #9 AND #10 | 228 |

| Cochrane Library | | |
| --- | --- | --- |
| # | Query | Results |
| 1 | MeSH descriptor: [Acquired Immunodeficiency Syndrome] explode all trees | 2522 |
| 2 | MeSH descriptor: [HIV] explode all trees | 4167 |
| 3 | #1 OR #2 | 6385 |
| 4 | (immunodeficiency associated virus OR Human Immunodeficiency Viruses OR human immunodeficiency virus infection OR Human immuno deficiency virus OR HIV OR aquired immunodeficiency syndrome OR aquired immune deficiency syndrome OR AIDS Viruses OR aids related virus OR aids associated virus OR aids associated retrovirus OR aids associated lentivirus OR AIDS OR acquired immunodeficiency virus syndrome OR Acquired Immuno-Deficiency Syndromes OR Acquired Immunodeficiency Syndromes OR Acquired Immunodeficiency Syndrome Virus OR acquired immunodeficiency disease syndrome OR Acquired Immune Deficiency Syndrome Virus OR Acquired Immune Deficiency Syndrome OR acquired immune deficiency disease syndrome OR acquired human immunodeficiency syndrome):ti,ab,kw | 41268 |
| 5 | #3 OR #4 | 41307 |
| 6 | (Smok* OR drink* OR alcohol):ti,ab,kw | 91292 |
| 7 | #5 AND #6 | 2761 |
| 8 | (randomized controlled trial OR controlled clinical trial OR placebo OR random allocation OR randomized):ti,ab,kw | 1438275 |
| 9 | #7 AND #8 | 1998 |
| 10 | (digital OR telephone OR telemedicine OR internet OR web OR application OR message or phone or mobile or remote or website):ti,ab,kw | 199154 |
| 11 | #9 AND #10 | 405 |

| Web of science | | |
| --- | --- | --- |
| # | Query | Results |
| 34 | immunodeficiency associated virus OR Human Immunodeficiency Viruses OR human immunodeficiency virus infection OR Human immuno deficiency virus OR HIV OR aquired immunodeficiency syndrome OR aquired immune deficiency syndrome OR AIDS Viruses OR aids related virus OR aids associated virus OR aids associated retrovirus OR aids associated lentivirus OR AIDS OR acquired immunodeficiency virus syndrome OR Acquired Immuno-Deficiency Syndromes OR Acquired Immunodeficiency Syndromes OR Acquired Immunodeficiency Syndrome Virus OR acquired immunodeficiency disease syndrome OR Acquired Immune Deficiency Syndrome Virus OR Acquired Immune Deficiency Syndrome OR acquired immune deficiency disease syndrome OR acquired human immunodeficiency syndrome (Topic) | 1217265 |
| 35 | ICT EXPRESS (Publication Titles) and 2025 or 2024 or 2023 (Publication Years) and Article or Early Access or Review Article (Document Types) | 424 |
| 36 | Smok* OR drink* OR alcohol (Topic) | 1248039 |
| 38 | randomized controlled trial OR controlled clinical trial OR placebo OR random allocation OR randomized (Topic) | 1494207 |
| 39 | TSINGHUA SCIENCE "AND" TECHNOLOGY (Publication Titles) and 2025 or 2024 or 2023 (Publication Years) and Article or Early Access or Review Article (Document Types) | 388 |
| 40 | digital OR telephone OR telemedicine OR internet OR web OR application OR message or phone or mobile or remote or website (Topic) | 9054265 |
| 41 | #34 AND #38 AND #36 AND #40 | 546 |
|  |  |  |
