# Supplementary material for: Effects of telehealth-integrated interventions for smoking cessation and alcohol reduction among people living with HIV: a systematic review and meta-analysis
Source: Front Public Health. 2026 Oct 23;14:1962757. doi: 10.3389/fpubh.2026.1962757 (PMC13739805; doi:10.3389/fpubh.2026.1962757)
Supplement: Supplementary file 2 [file Data_Sheet_2.DOCX]

**Appendix 2: Evaluation Table for the Quality of GRADE Evidence**

| **Certainty assessment** | | | | | | | **Certainty** |
| --- | --- | --- | --- | --- | --- | --- | --- |
| **№ of studies** | **Study design** | **Risk of bias** | **Inconsistency** | **Indirectness** | **Imprecision** | **Publication bias** |  |
| **7-day point prevalence abstinence rate** | | | | | | | |
| 11 | RCT studies | not serious | not serious | not serious | not serious | not serious | ⨁⨁⨁⨁ High |
| **30-day point prevalence abstinence rate** | | | | | | | |
| 3 | RCT studies | not serious | not serious | not serious | Serious^1^ | Serious^2^ | ⨁⨁◯◯ Low |
| **number of drinking days in the past 30 days** | | | | | | | |
| 3 | RCT studies | not  serious | not serious | not serious | Serious^3^ | Serious^2^ | ⨁⨁◯◯ Low |
| **drinks per drinking day** 3 \| RCT studies \| \| not serious \| not serious \| not serious \| Serious1 \| Serious2 \| ⨁⨁◯◯ Low | | | | | | | |

**CI:** confidence interval

Explanations

1.The number of included studies was small, and the quality of the evidence was downgraded.

2.The number of included studies was relatively small, and thus a detection of publication bias could not be conducted.

3.The confidence interval crosses the null line (0), suggesting that telehealth may have no effect or only a minimal effect on this outcome.
